# Associations between long-term fine particulate matter exposure and hospital procedures in heart failure patients

**DOI:** 10.1371/journal.pone.0283759

**Published:** 2023-05-03

**Authors:** Samantha Catalano, Joshua Moyer, Anne Weaver, Qian Di, Joel D. Schwartz, Michael Catalano, Cavin K. Ward-Caviness

**Affiliations:** 1 Department of Biology, University of North Carolina at Chapel Hill, Chapel Hill, North Carolina, United States of America; 2 Center for Public Health and Environmental Assessment, US Environmental Protection Agency, Chapel Hill, North Carolina, United States of America; 3 Research Center for Public Health, School of Medicine, Tsinghua University, Beijing, China; 4 Harvard TH Chan School of Public Health, Boston, Massachusetts, United States of America; 5 Division of Cardiovascular Surgery, Department of Surgery, Hospital of the University of Pennsylvania, Philadelphia, Pennsylvania, United States of America; Kyung Hee University School of Medicine, REPUBLIC OF KOREA

## Abstract

**Background:**

Ambient fine particulate matter (PM_2.5_) contributes to global morbidity and mortality. One way to understand the health effects of PM_2.5_ is by examining its impact on performed hospital procedures, particularly among those with existing chronic disease. However, such studies are rare. Here, we investigated the associations between annual average PM_2.5_ and hospital procedures among individuals with heart failure.

**Methods:**

Using electronic health records from the University of North Carolina Healthcare System, we created a retrospective cohort of 15,979 heart failure patients who had at least one of 53 common (frequency > 10%) procedures. We used daily modeled PM_2.5_ at 1x1 km resolution to estimate the annual average PM_2.5_ at the time of heart failure diagnosis. We used quasi-Poisson models to estimate associations between PM_2.5_ and the number of performed hospital procedures over the follow-up period (12/31/2016 or date of death) while adjusting for age at heart failure diagnosis, race, sex, year of visit, and socioeconomic status.

**Results:**

A 1 μg/m^3^ increase in annual average PM_2.5_ was associated with increased glycosylated hemoglobin tests (10.8%; 95% confidence interval = 6.56%, 15.1%), prothrombin time tests (15.8%; 95% confidence interval = 9.07%, 22.9%), and stress tests (6.84%; 95% confidence interval = 3.65%, 10.1%). Results were stable under multiple sensitivity analyses.

**Conclusions:**

These results suggest that long-term PM_2.5_ exposure is associated with an increased need for diagnostic testing on heart failure patients. Overall, these associations give a unique lens into patient morbidity and potential drivers of healthcare costs linked to PM_2.5_ exposure.

## Introduction

Long-term exposure to ambient particulate matter smaller than 2.5 μm in diameter (PM_2.5_) has been shown to increase mortality and morbidity. PM_2.5_ can come from a variety of sources such as combustion, blown dust, industry activities, and traffic. PM_2.5_ is also formed via secondary chemical reactions of primary emitted pollutants [1]. Despite global efforts to reduce particulate matter concentrations, ambient air pollution remains a substantial contributor to the global burden of disease [2]. There are several proposed underlying mechanisms through which PM_2.5_ causes adverse health effects. The larger sized fractions of PM_2.5_ are not readily transported into the circulatory system and are instead most often linked to the triggering of systemic inflammation from the lungs and other intermediate pathways, e.g. lipid oxidation, that can cause extra-pulmonary effects. For example, at the molecular level, PM_2.5_ inhalation can increase the intracellular levels of reactive oxygen species, which causes pulmonary oxidative stress; this can instigate systemic inflammatory responses at the physiological level [3, 4]. The smallest sized fractions of PM_2.5_, often called ultrafine particulates, have shown an ability to translocate out of the lungs where they may exert direct toxicity on the heart, kidneys, and other organ systems [5].

These contributions of PM_2.5_ to global morbidity and mortality are exacerbated by aging populations and an increasing prevalence of chronic conditions such as heart failure (HF) [6]. It is estimated that by 2030, 1 in every 33 people in the United States will suffer from HF [7]. Moreover, the total direct medical costs of HF are projected to increase from $21 billion in 2012 to $53 billion in 2030 [7].

Hospital procedures—which we defined as any billable task or service a medical practitioner may provide to a patient—contribute to healthcare costs. Further, an increased number of non-elective hospital procedures may indicate worsening health in the individual and population. Long-term exposure to particulate matter is associated with multiple health outcomes, but it has never been investigated in relation to the performance of hospital procedures on a patient population. Quantifying the relationship between elevated concentrations of ambient particulate matter and hospital procedures would provide further evidence of the impact of poor air quality on individual health. Since hospital procedures are an indicator of patient morbidity and a driver of medical costs, and air pollution has been found to have a close temporal association with hospitalizations, studies investigating the associations between air pollution and hospital procedures may also present a new lens into understanding air pollution-associated health effects and the impacts of environmental exposures on the economics of the healthcare industry [8].

Here we estimate the associations between the frequencies of specific hospital procedures performed and the annual-average PM_2.5_ exposures among HF patients. Long-term PM_2.5_ exposure has been associated with increased mortality, hospitalizations, and readmissions among HF patients [9, 10], but this will be the first study to examine the impact of air pollution on performed hospital procedures in this understudied patient population. We hypothesize that long-term exposure to air pollution is associated with increased performance of hospital procedures among HF patients.

## Methods

### Study cohort

This study used the US Environmental Protection Agency (EPA) Clinical and Archived records Research for Environmental Studies (CARES) resource, which has been used in previous studies of environmental health among HF patients [9, 10]. EPA CARES is a resource that merges electronic health records from the University of North Carolina Healthcare System (UNCHCS) with environmental exposure data. This study was approved by the institutional review board of the University of North Carolina–Chapel Hill (IRB 17–0150), and informed consent was waived for this analysis of existing health records. For this study, we used electronic health records from patients with a diagnosis of HF between July 1, 2004 and December 31, 2016, which has been used in previous studies of mortality and hospital admissions [9, 10]. Patients for this HF cohort had to be aged 20 or older at the time of first HF diagnosis (to remove cases likely due to congenital disease) and had to have a valid address listed in their electronic health records for their date of HF diagnosis (to determine environmental exposures) [9]. We further required that individuals have at least one hospital visit prior to their HF diagnosis to limit the impact of those who were diagnosed at another hospital system and later seen within a UNCHCS hospital or clinic [10]. HF was defined according to the International Classification of Diseases, Ninth Revision (ICD‐9) codes 428.x and the International Classification of Diseases, Tenth Revision (ICD‐10) codes I50.x [9]. Electronic health records for this population included demographics, clinical variables such as address history, dates of procedures, and dates of diagnoses (via ICD-9 and ICD-10 codes). For this study environmental exposures were linked to individuals based on their date of HF diagnosis and the associated address from their electronic health record allowing us to see how environmental exposures at this critical time period impacted long-term health as reflected in hospital procedures. Census block group-level socioeconomic data from the 2010 census—chosen as it was the approximate midpoint of the study observation period—was used to evaluate area-level socioeconomic status based on geocoded primary address, as information on individual-level socioeconomic status is missing from the electronic health record. Individuals were considered successfully geocoded with a minimum of zip code level geocoding.

Individuals who did not receive any of the common (defined as >10% in frequency, as detailed in the Procedures section of the Methods below) procedures over their follow-up time may have represented a distinct set of (potentially healthier) patients or those who received healthcare at multiple hospital systems not in communication with the UNCHCS and, thus, they were removed from primary analyses—though we conducted sensitivity analyses to examine the impact of this choice.

### Air pollution data

PM_2.5_ concentrations were estimated at 1x1 km resolution from 2000–2016 using a hybrid model that incorporated aerosol optical depth, ground-based monitoring, chemical transport models, land use, and meteorology [11]. Study participants were linked to PM_2.5_ data based on their geocoded primary residence at the date of HF diagnosis. Daily PM_2.5_ was averaged to create an annual average estimate based on the 365 days of exposure prior to their HF diagnosis.

### Procedures

Out of all the observed procedures, we removed any “procedure” that was simply a hospital visit or a follow-up of a previous procedure. The data was further limited to only those procedures that were seen in at least 10% of the population to allow for a sufficient sample size for primary and subgroup analyses.

To eliminate potential duplicate procedure entries, we aggregated procedures to the day of their occurrence—effectively changing them into an indicator of whether the procedure was performed on that patient on a particular day—and merged procedures that changed names over the study period using the Current Procedure Terminology (CPT) code, which was static in our dataset over the study timeframe. We observed that tests classified as stress tests, including cardiovascular stress tests with and without report, technetium tc-99m sestamibi, and regadenoson injections, generally co-occurred. We thus combined these stress tests into one procedure category termed “stress tests” and treated them as a single procedure for the analyses. After all data merging and cleaning, there were 53 distinct procedures (S1 Table in S1 File). These procedures were primarily routine and diagnostic, likely due to the decision to only include procedures seen in at least 10% of the population.

### Statistical analysis

We used a quasi-Poisson regression model (which allows for over or under dispersion) to model the association between annual average PM_2.5_ exposure at the time of HF diagnosis and the procedure count for each of the 53 procedures over the follow-up observation period (12/31/2016 or date of death) [12]. Confounders were selected based on previous studies done in this population and included individual level confounders as well as area-level (census block group) confounders to control for urbanicity and area-level socioeconomic status [9, 10]. Individual-level confounders were age at HF diagnosis; race; sex; and smoking status. Census block group confounders were based on the 2010 census and were median income; median household value; year of HF diagnosis; and the percentage of the census block that is urban, on public assistance, or below the poverty line. The screening mammography was the only sex-specific procedure and thus not adjusted for sex. The primary exposure for all models was the 365-day average (annual average) PM_2.5_ based on the day of HF diagnosis, and the log of the follow-up time was included as an offset in the models. The outcome was the total number of days on which a given procedure was performed for each patient over the follow-up time.

We further examined the robustness of the associations to confounder inclusion by additionally adjusting for access to healthcare and healthy foods, both of which may be related to socioeconomic status and potentially confound responses to air pollution. To perform this adjustment, we utilized county-level data on the number of primary care physicians, dentists, mental health providers, and non-primary care physicians per 100,000 people, along with average annual healthcare cost and food environment index from the 2015 County Health Rankings [13]. The food environment index is a scaled index ranging from 0 (worst) to 10 (best) that incorporates both limited access to healthy foods and food insecurity using data from the United States Department of Agriculture [14, 15].

We then performed a series of sensitivity analyses to understand the robustness of the significant associations from our primary analyses. We examined a Poisson mixed-effects model with a random intercept for residential zip code. The model was adjusted for the same variables as the original quasi-Poisson regression (including the offset term). To improve convergence, continuous variables were standardized to a mean of zero and a standard deviation of one.

Excluding patients who did not receive any procedure may have introduced bias if the patients who were excluded substantially differed from those who were included. We examined this potential source of bias by including all patients and using a zero-inflated Poisson model to account for the number of patients who did not receive procedures. The confounder adjustment for the zero-inflated Poisson model remained the same as the primary analysis.

To examine if results differed among those exposed to PM_2.5_ less than the current national ambient air quality standard, we restricted our study cohort to those with an annual average PM_2.5_ < 12 μg/m^3^ and reran our primary analysis on this restricted cohort [16].

We also examined population strata based on age, sex, race, urbanicity, and median household income to observe if there were strata-specific associations. All stratified models were run only on those procedures identified as Bonferroni significant in the primary analyses. The subgroups for age included less than 50 years, 50 to 65 years, and greater than or equal to 65 years. Racial subgroups were defined as the Black and White patients, as there were not enough in the “other” racial category for analyses. Urbanicity was determined using the percentage of the census block group based on the 2010 census; urban areas were defined as 100% urban (which captured slightly more than the top third of the distribution), rural areas were defined as 0% urban (which captured slightly less than the bottom third of the distribution), and all other areas were defined as 1%-99% urban, which we call suburban (though many are quite rural or urban). The subgroups for median household income were above and below the median income of $49,355.

To acknowledge the competing risk of death, we performed a sensitivity analysis where observations were weighted using stabilized inverse probability weights for the risk of death, as done in similar studies [10, 17]. The confounder adjustment remained the same as for the primary analysis.

All analyses were run in R version 4.0.2 [18]. Associations were converted to the percent change in outcome by exponentiating the regression coefficient, subtracting one, and multiplying the result by 100. All results are given as the percent change and associated 95% confidence interval (CI) per 1 μg/m^3^ increase in PM_2.5_. Statistical significance for the associations was set at p < 0.05/53, reflecting a Bonferroni multiple test adjustment for the number of procedures examined.

## Results

The final study cohort consisted of 15,979 patients with an average follow-up time of 2.94 years (Table 1, S1 Fig in S1 File). Procedure frequencies per patient were consistent across the major demographic categories of age, race, and sex (Table 2).

**Table 1:** Demographic variables (N = 15,979).

	n	%
Race: Black	4,394	27.50%
Race: White	10,568	66.14%
Race: Other	1,017	6.36%
Sex: Female	8,442	52.83%
Sex: Male	7,537	47.17%
Smoking Status: Current	1,721	10.77%
Smoking Status: Former	5,406	33.83%
Smoking Status: Never	5,021	31.42%
Smoking Status: Unknown	3,831	23.98%
Chronic Kidney Disease	10,388	65.01%
Ischemic Heart Disease	10,515	65.81%
Hypertension	13,008	81.41%
Chronic Obstructive Pulmonary Disease	7,202	45.07%
Type 2 Diabetes	5,978	37.41%
Dyslipidemia	13,301	83.24%
Peripheral Arterial Disease	7,344	45.96%
	**mean**	**SD**
Age (years)	68.07	14.75
Annual Average PM_2.5_ Exposure (μg/m^3^)	9.72	1.55
Median Income ($)	54,441.88	26,547.82
Median House Value ($)	183,591.38	108,266.63
% on Public Assistance	1.99	2.97
% Urban	63.09	42.05
% Below the Poverty Line	16.99	14.01

Shown in this table are the demographic variables for the 15,979 patients with at least 1 procedure. Percent on public assistance, percent urbanicity, and percent below the poverty line were taken from census block group level estimates from the 2010 Census.

**Table 2:** Number of procedures per patient.

Patient Population	median	IQR	SD
Entire Cohort	6	15	20.50
Black	7	16	23.98
White	6	14	18.86
Other Race	5	11	19.90
Female	6	14	20.47
Male	7	15	20.53
< 50 years old	9	19	29.78
< 65 years old	8	17	25.31
≥ 65 years old	6	13	16.29

The number of procedures per patient in the entire cohort and stratified by race, sex, and age.

We also examined the 4,941 patients out of the original patient cohort who did not receive any of the 53 procedures (S2 Table in S1 File). This patient population was slightly older, less sick in terms of co-existing conditions, and consisted of fewer smokers and former smokers. While no major differences in their demographics were observed, we cannot rule out unobserved systematic differences between these populations.

**Fig 1:**
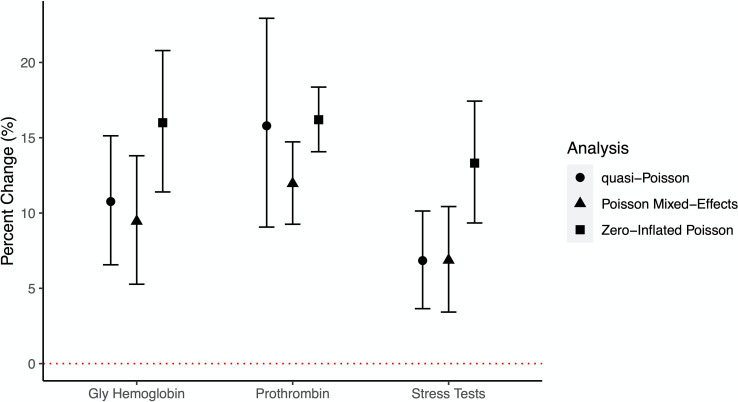
Associations between annual average PM_2.5_ and hospital procedures among heart failure patients. Shown here are associations between annual average PM_2.5_ and the three Bonferroni significant procedures. Significance was determined according to the primary analysis approach (quasi-Poisson). We also show the percent change and 95% confidence interval associated with the sensitivity analysis for the Poisson Mixed-Effects model (which included a random intercept for zip code) and Zero-Inflated Poisson model (which included all individuals as opposed to just those with at least 1 procedure). See Methods for complete description of all analysis approaches. Percent change and 95% confidence interval presented per 1 μg/m^3^ increase in PM_2.5_. Gly Hemoglobin = Glycosylated Hemoglobin Test; Prothrombin = Prothrombin Time Test.

In our primary analyses three procedures were significant after multiple test correction: glycosylated hemoglobin tests (10.8% increase; 95% CI = 6.56%, 15.1%); prothrombin time tests (15.8% increase; 95% CI = 9.07%, 22.9%); and stress tests (6.84% increase; 95% CI = 3.65%, 10.1%) (Fig 1, Table 3, S3 Table in S1 File). Associations for all three Bonferroni significant procedures were attenuated after further adjustment for county-level access to healthcare and healthy foods, though associations with prothrombin time tests remained significant (1.46% increase; 95% CI = 0.71%, 2.22%; S4 Table in S1 File).

**Table 3:** Significant procedure descriptions.

Procedure	Procedure Code	Procedure Description
**Glycosylated Hemoglobin Test**	83036	measures HbA1c, which determines the average blood glucose level for the 2 to 3 months before the test; often used to monitor diabetes
**Prothrombin Time Test**	85610	measures how long it takes blood to clot; often used to help detect and diagnose a bleeding disorder or excessive clotting disorder
**Stress Tests**	*Stress Tests	measures the heart’s ability to respond to external stress in a controlled clinical environment; includes both exercise stress tests and pharmacologic stress tests

The Procedure Codes are Current Procedural Terminology (CPT codes) for the three significant procedures, which are developed, maintained, and copyrighted by the American Medical Association (AMA) [29]. *The “Stress Tests” procedure as defined by this study (see Methods) included Cardiovascular Stress Test with Interpretation and Report (93015), Cardiovascular Stress Test without Interpretation and Report (93016), Technetium Tc-99m Sestamibi (A9500), and Regadenoson Injection (J2785).

As there may be clustering of environmental and sociodemographic characteristics by zip code, we ran a Poisson mixed-effects model with a random intercept for zip code. Associations with the three significant procedures remained unchanged (Fig 1). Associations also remained similar when the patients who did not undergo each of the 53 procedures examined were added into the analysis cohort (Fig 1, S4 Table in S1 File). Given the strong mortality risk among HF patients, the risk of death may present an important competing risk to account for. Using inverse probability weighting, as described in the Methods, we adjusted for the competing risk of death, and observed no differences in the associations as compared to the primary analyses described above (S2 Fig in S1 File).

To understand if associations differed in areas with lower ambient PM_2.5_ concentrations, we examined associations for all individuals whose annual average PM_2.5_ exposure was less than the current PM_2.5_ annual average standard (National Ambient Air Quality Standard) of 12 μg/m^3^. Among this population, associations between PM_2.5_ and prothrombin time tests remained the same (15.1% increase; 95% CI = 8.07%, 22.7%), while associations between PM_2.5_ and glycosylated hemoglobin tests were attenuated (5.98% increase; 95% CI = 1.33%, 10.8%). No association between annual average PM_2.5_ and stress tests were seen amongst those with exposures less than 12 μg/m^3^ (S4 Table in S1 File).

We examined potential strata-specific association differences based on age, sex, race, urbanicity, and median household income of the census block group for the three Bonferroni significant procedures. For glycosylated hemoglobin tests, associations were stronger among Black study participants than White participants—and associations were stronger in residents of the suburban and urban census block groups than in those of the rural census block group. However, the wide, overlapping confidence intervals of these associations preclude any strong statements on strata-specific associations (S3-S5 Figs in S1 File).

## Discussion

We examined associations between annual average PM_2.5_ exposure and the occurrence of hospital procedures in HF patients and observed three statistically significant associations, all of which are related to diagnostic testing for cardiorespiratory health.

Exposure to particulate matter is associated with altered glucose metabolism and increased risk of diabetes [19]. We observed statistically significant associations between glycosylated hemoglobin (HbA1C) tests and annual average PM_2.5_ exposure. Glycosylated hemoglobin tests are reflective of a patient’s blood sugar concentrations throughout the previous 3 months, and they may be used to both screen for diabetes in high-risk individuals and monitor progression of diabetes [20]. Elevated ambient concentrations of PM_2.5_ may contribute to elevated blood glucose and prevalence of diabetes, and, thus, an increased need for glycosylated hemoglobin tests [21, 22].

The other two significant procedures—stress tests and prothrombin time tests—are related to cardiovascular testing. In this study, “stress tests” included cardiovascular stress tests with and without interpretation and report, technetium sestamibi radiological imaging, and regadenoson injections, thus encompassing both exercise stress tests and pharmacologic stress tests [23]. Exercise and pharmacologic stress tests can help physicians understand the cause and severity of a HF patient’s condition or evaluate and modify their treatment plans. They also are often used in the diagnosis, workup, and management planning of ischemic heart disease and valvular heart disease [24, 25]. Since PM_2.5_ exposure is a risk factor for cardiovascular morbidity and mortality, these stress tests may be more necessary for patients with increased morbidity due to higher long-term exposure to PM_2.5_ [26].

Prothrombin time tests measure how long blood takes to clot and are often used to help detect and diagnose a bleeding disorder or excessive clotting disorder. Hematological aberrations are associated with long-term exposure to PM_2.5_, the mechanism of which may be related to increased inflammatory cytokine levels, oxidative stress, platelet activation, stimulated coagulation pathway, and reduced fibrinolysis [27]. Thus, disrupted hemostasis due to long-term PM_2.5_ exposure may increase the need for prothrombin time tests.

Associations were robust to a range of modeling scenarios (S4 Table in S1 File). Associations with prothrombin time tests were unchanged when restricted to exposures less than the current national standards, while associations with glycosylated hemoglobin were approximately halved and associations with stress tests attenuated towards the null. As each test may point towards a different underlying biological mechanism (hemostasis, cardiovascular function, and glucose metabolism), these differences may give clues as to the different mechanisms of increased morbidity that may be seen among HF patients as the concentration (and potentially composition) of ambient PM_2.5_ changes. Importantly, all associations were substantially attenuated when additionally adjusting for county-level indicators of access to healthcare and healthy food options, suggesting that these variables may play a role in moderating associations with annual average PM_2.5_ (S4 Table in S1 File). Formal examinations of the social and built environment factors that may moderate these associations were outside the scope of this manuscript and would be best performed with more comprehensive and geospatially resolved maps of these factors.

The strengths of our approach include the examination of HF patients, a vulnerable patient population that is more likely to have severe health outcomes from environmental exposures. Despite this vulnerability, HF patients have been understudied with respect to their environmental health risks. Another strength of this study was the large sample size and up to 12 years of follow-up. This study also used high spatial resolution (1x1 km) of the air pollution exposure data and patient address data, including street level geocoding for most patients and no coarser than zip code level geocoding.

Our approach also had a variety of limitations. One limitation is that the electronic health records do not include all possible confounders; in particular, individual-level socioeconomic status was missing. We used data from the US census to adjust for area-level socioeconomic status, which is a widely utilized approach when individual-level data is missing. As we only used records from UNCHCS, procedures that were not performed at an affiliated hospital or clinic were not captured in our data unless those records were transferred to the UNCHCS electronic health record system; further, these associations may not generalize other healthcare systems, such as those with a more rural catchment area. However, previous associations utilizing just the UNCHCS HF patient population have shown concordance with studies done utilizing the entire southeastern United States, so this analysis potentially has at least regional generalizability [10]. Finally, while we used air pollution exposure data with a high spatial resolution, the exposures were ultimately determined using the primary addresses of the patients. While this is standard in the field, reflecting that many people spend most of their time at their primary residence, it may not completely capture the PM_2.5_ exposure of individuals.

Moving forward, it will be important to investigate short-term exposures alongside long-term exposures. Future studies should also evaluate other patient populations and consider exposures beyond PM_2.5_ total mass, including the examination of different air pollutants such as ozone, ultrafine particulates/particle number count, and the chemical composition of PM_2.5_. Finally, this study did not have the scope to connect performed hospital procedures with the broader economics of hospital system operations. However, given that PM_2.5_ is estimated to cost the US economy billions of dollars annually, quantifying the economic impact of PM_2.5_ through the lens of performed hospital procedures might provide unique insights that can help quantify the economic impacts of PM_2.5_ exposure as well as the health, wellness, and morbidity impacts [28].

## Conclusions

We observed that elevated long-term exposure to PM_2.5_ is associated with the increased performance of glycosylated hemoglobin tests, prothrombin time tests, and stress tests among HF patients. HF patients presented a unique patient group to examine this important question, as they have both increased environmental sensitivity and high rates of hospital utilization. This study adds to the field by quantifying PM_2.5_ impacts on performed hospital procedures, thereby allowing researchers to better understand air pollution health effects and helping to provide key information needed to estimate the total burden of PM_2.5_ on both patients and hospital systems.

## Supporting information

S1 File (DOCX)
