# Supplementary material for: Associations between long-term fine particulate matter exposure and hospital procedures in heart failure patients
Source: PLoS One. 2023 May 3;18(5):e0283759. doi: 10.1371/journal.pone.0283759 (PMC10155991; doi:10.1371/journal.pone.0283759)
Supplement: S1 File — (DOCX) [file pone.0283759.s001.docx]

**Supporting Information**

**Tables**

**S1 Table. All 53 Procedures and their CPT Codes**

| Procedure | Procedure Code |
| --- | --- |
| 12-Lead Electrocardiogram | 3120F |
| Insertion of Non-tunneled Centrally Inserted Central Venous Catheter, Age 5 Years or Older | 36556 |
| Head/Brain CT Scan, without Contrast | 70450 |
| Brain (Including Brain Stem) MRI, without then with Contrast | 70553 |
| Chest X-Ray, Single View | 71010 |
| Chest X-Ray, Two Views | 71020 |
| CT of the Thorax, without Contrast | 71250 |
| CT of the Thorax, with Contrast | 71260 |
| Computed Tomography Angiography | 71275 |
| Radiologic Examination, Spine, Lumbosacral | 72100 |
| Radiologic Examination, Foot | 73630 |
| Abdomen X-Ray, Single View | 74000 |
| CT of the Abdomen, with Contrast | 74160 |
| CT of the Abdomen and Pelvis, with Contrast | 74177 |
| Ultrasound, Abdominal, Real-Time with Image Documentation | 76700 |
| Ultrasound, Retroperitoneal, Real-Time with Image Documentation | 76770 |
| Ultrasound Guidance Procedures | 76937 |
| Fluoroscopic Guidance | 77001 |
| Dual-Energy X-Ray Absorptiometry, Bone Density Study, 1 or More Sites | 77080 |
| Myocardial Perfusion Imaging, Tomographic (SPECT) | 78452 |
| Basic Metabolic Panel | 80048 |
| Comprehensive Metabolic Panel | 80053 |
| Lipid Panel | 80061 |
| Urinalysis, by Dip Stick or Tablet Reagent | 81002 |
| Glycosylated Hemoglobin Test | 83036 |
| Protein, Electrophoretic Fractionation and Quantification, Serum | 84165 |
| Thyroid Stimulating Hormone Assay | 84443 |
| Complete Blood Count with Automated Differential WBC Count | 85025 |
| Prothrombin Time Test | 85610 |
| Immunofixation Electrophoresis | 86334 |
| Cytopathology Procedures | 88112 |
| Surgical Pathology, Gross and Microscopic Examination | 88307 |
| Immunohistochemistry or Immunocytochemistry, per Specimen | 88342 |
| Vaccine Administration | 90471 |
| Electrocardiogram | 93000 |
| Electrocardiogram, Routine ECG with at least 12 Leads | 93010 |
| Echocardiogram, Complete with Doppler | 93306 |
| Echocardiography, Transthoracic, Real-Time with Image Documentation (2D), includes M-Mode Recording | 93307 |
| Echocardiography, Transthoracic, Limited | 93308 |
| Echocardiogram, Complete with Color Doppler | 93325 |
| Cardiac Catheterization | 93458 |
| Duplex Scan of Extracranial Arteries, Bilateral | 93880 |
| Limited Bilateral Noninvasive Physiologic Studies of Upper and Lower Extremity Arteries | 93922 |
| Duplex Scan of Extremity Veins, Bilateral | 93970 |
| Duplex Scan of Extremity Veins, Unilateral or Limited | 93971 |
| Spirometry, including Graphic Record, Total and Timed Vital Capacity, Expiratory Flow Rate Measurement(s), with or without Maximal Voluntary Ventilation | 94010 |
| Bronchodilation Responsiveness, Pre- and Post-Bronchodilator Administration | 94060 |
| Diffusing Capacity | 94729 |
| New or Established Patient Initial Hospital Observation Care Services | 99220 |
| Administration of Influenza Virus Vaccine | G0008 |
| Screening Mammography, Bilateral, including Computer-Aided Detection | G0202 |
| Prescription(s) Generated and Transmitted via a Qualified ERX System | G8553 |
| Stress Tests | *Stress Tests |

Procedures selected were those with at least 10% of the population having the procedure at least once during the observation period. The Procedure Codes are Current Procedural Terminology (CPT codes), which are developed, maintained, and copyrighted by the American Medical Association (AMA) [29]. *The code for Stress Tests was determined within this study and included Cardiovascular Stress Test with Interpretation and Report (93015), Cardiovascular Stress Test without Interpretation and Report (93016), Technetium Tc-99m Sestamibi (A9500), and Regadenoson Injection (J2785).

**S2 Table. Demographic Variables (N = 4,941)**

|  | **n** | **%** |
| --- | --- | --- |
| Race: Black | 1,170 | 23.68% |
| Race: White | 3,307 | 66.93% |
| Race: Other | 464 | 9.39% |
| Sex: Female | 2,556 | 51.73% |
| Sex: Male | 2,385 | 48.27% |
| Smoking Status: Current | 308 | 6.23% |
| Smoking Status: Former | 1,109 | 22.44% |
| Smoking Status: Never | 1,155 | 23.38% |
| Smoking Status: Unknown | 2,369 | 47.95% |
| Chronic Kidney Disease | 2,795 | 56.57% |
| Ischemic Heart Disease | 2,923 | 59.16% |
| Hypertension | 3,594 | 72.74% |
| Chronic Obstructive Pulmonary Disease | 1,823 | 36.90% |
| Type 2 Diabetes | 1,649 | 33.37% |
| Dyslipidemia | 3,741 | 75.71% |
| Peripheral Arterial Disease | 1,885 | 38.15% |
|  | **mean** | **SD** |
| Age (years) | 71.01 | 14.92 |
| Annual Average PM_2.5_ Exposure (µg/m^3^) | 10.36 | 2.21 |
| Median Income ($) | 53,418.08 | 25,733.97 |
| Median House Value ($) | 180,003.52 | 106,840.53 |
| % on Public Assistance | 1.97 | 2.96 |
| % Urban | 65.35 | 41.53 |
| % Below the Poverty Line | 17.12 | 13.89 |

Shown in this table are the demographic variables for the 4,941 patients who did not receive a procedure. Percent on public assistance, percent urbanicity, and percent below the poverty line were taken from census block group level estimates from the 2010 Census.

**S3 Table. quasi-Poisson regression results for all 53 procedures**

| Procedure | Percent Change | Lower Bound of 95% Confidence Interval | Upper Bound of 95% Confidence Interval | p-value | Bonferroni-Adjusted  p-value |
| --- | --- | --- | --- | --- | --- |
| Insertion of Non-tunneled Centrally Inserted Central Venous Catheter, Age 5 Years or Older | -3.73% | -21.4% | 17.8% | 0.71 | 1 |
| Echocardiography, Transthoracic, Real-Time with Image Documentation (2D), includes M-Mode Recording | 2.54% | -15.3% | 24.1% | 0.80 | 1 |
| Immunofixation Electrophoresis | -5.47% | -21.8% | 14.3% | 0.56 | 1 |
| Protein, Electrophoretic Fractionation and Quantification, Serum | -6.57% | -21.3% | 10.9% | 0.44 | 1 |
| Computed Tomography Angiography | -1.44% | -16.4% | 16.2% | 0.86 | 1 |
| Surgical Pathology, Gross and Microscopic Examination | -10.38% | -24.1% | 5.83% | 0.20 | 1 |
| Immunohistochemistry or Immunocytochemistry, per Specimen | 1.37% | -13.5% | 18.8% | 0.87 | 1 |
| Cytopathology Procedures | 0.38% | -13.6% | 16.6% | 0.96 | 1 |
| CT of the Abdomen, with Contrast | -4.51% | -17.4% | 10.3% | 0.53 | 1 |
| Ultrasound, Abdominal, Real-Time with Image Documentation | -1.92% | -14.7% | 12.8% | 0.79 | 1 |
| Brain (Including Brain Stem) MRI, without then with Contrast | -7.66% | -19.6% | 6.11% | 0.26 | 1 |
| CT of the Thorax, with Contrast | -1.11% | -13.7% | 13.3% | 0.87 | 1 |
| CT of the Abdomen and Pelvis, with Contrast | -5.85% | -17.7% | 7.63% | 0.38 | 1 |
| Fluoroscopic Guidance | -1.90% | -14.3% | 12.3% | 0.78 | 1 |
| Spirometry, including Graphic Record, Total and Timed Vital Capacity, Expiratory Flow Rate Measurement(s), with or without Maximal Voluntary Ventilation | 1.05% | -10.8% | 14.4% | 0.87 | 1 |
| Ultrasound Guidance Procedures | -7.74% | -18.6% | 4.53% | 0.21 | 1 |
| Abdomen X-Ray, Single View | -6.06% | -16.5% | 5.69% | 0.30 | 1 |
| CT of the Thorax, without Contrast | -5.26% | -15.9% | 6.70% | 0.37 | 1 |
| Radiologic Examination, Foot | -10.38% | -20.1% | 0.52% | 0.06 | 1 |
| Chest X-Ray, Single View | -11.04% | -19.5% | -1.64% | 0.02 | 1 |
| Cardiac Catheterization | -7.17% | -16.0% | 2.61% | 0.15 | 1 |
| Ultrasound, Retroperitoneal, Real-Time with Image Documentation | -5.86% | -14.7% | 3.92% | 0.23 | 1 |
| Bronchodilation Responsiveness, Pre- and Post-Bronchodilator Administration | -0.60% | -9.60% | 9.29% | 0.90 | 1 |
| Radiologic Examination, Spine, Lumbosacral | -1.61% | -10.1% | 7.75% | 0.73 | 1 |
| Echocardiography, Transthoracic, Limited | -0.63% | -9.09% | 8.63% | 0.89 | 1 |
| Prescription(s) Generated and Transmitted via a Qualified ERX System | -2.26% | -10.2% | 6.36% | 0.60 | 1 |
| Echocardiogram, Complete with Color Doppler | 6.64% | -1.90% | 15.9% | 0.13 | 1 |
| 12-Lead Electrocardiogram | 3.40% | -4.46% | 11.9% | 0.41 | 1 |
| Head/Brain CT Scan, without Contrast | 2.26% | -5.13% | 10.2% | 0.56 | 1 |
| Limited Bilateral Noninvasive Physiologic Studies of Upper and Lower Extremity Arteries | 1.62% | -5.46% | 9.23% | 0.66 | 1 |
| Duplex Scan of Extremity Veins, Unilateral or Limited | 1.37% | -5.55% | 8.80% | 0.71 | 1 |
| New or Established Patient Initial Hospital Observation Care Services | 2.16% | -4.78% | 9.61% | 0.55 | 1 |
| Duplex Scan of Extremity Veins, Bilateral | 4.70% | -2.15% | 12.0% | 0.18 | 1 |
| Basic Metabolic Panel | 5.37% | -1.24% | 12.4% | 0.11 | 1 |
| Urinalysis, by Dip Stick or Tablet Reagent | 3.35% | -3.12% | 10.3% | 0.32 | 1 |
| Comprehensive Metabolic Panel | 5.17% | -1.11% | 11.8% | 0.11 | 1 |
| Complete Blood Count with Automated Differential WBC Count | 7.67% | 1.46% | 14.3% | 0.02 | 0.80 |
| Duplex Scan of Extracranial Arteries, Bilateral | 6.11% | 0.08% | 12.5% | 0.05 | 1 |
| Thyroid Stimulating Hormone Assay | -0.19% | -5.67% | 5.62% | 0.95 | 1 |
| Dual-Energy X-Ray Absorptiometry, Bone Density Study, 1 or More Sites | -0.23% | -5.43% | 5.27% | 0.93 | 1 |
| Diffusing Capacity | 1.03% | -4.25% | 6.60% | 0.71 | 1 |
| Electrocardiogram, Routine ECG with at least 12 Leads | -3.41% | -8.39% | 1.85% | 0.20 | 1 |
| Screening Mammography, Bilateral, including Computer-Aided Detection | -0.85% | -5.92% | 4.50% | 0.75 | 1 |
| Administration of Influenza Virus Vaccine | -1.30% | -6.03% | 3.66% | 0.60 | 1 |
| Lipid Panel | 2.49% | -1.93% | 7.10% | 0.27 | 1 |
| Vaccine Administration | 5.92% | 1.75% | 10.3% | 0.01 | 0.27 |
| Electrocardiogram | -2.00% | -5.78% | 1.94% | 0.32 | 1 |
| Myocardial Perfusion Imaging, Tomographic (SPECT) | 3.18% | -0.53% | 7.03% | 0.09 | 1 |
| Chest X-Ray, Two Views | -1.36% | -4.70% | 2.10% | 0.44 | 1 |
| Echocardiogram, Complete with Doppler | 2.20% | -0.72% | 5.20% | 0.14 | 1 |
| Prothrombin Time Test | 15.8% | 9.07% | 22.9% | 1.80E-06 | *9.54E-05 |
| Glycosylated Hemoglobin Test | 10.8% | 6.56% | 15.1% | 2.49E-07 | *1.32E-05 |
| Stress Tests | 6.84% | 3.65% | 10.1% | 1.98E-05 | *1.05E-03 |

The percent change, 95% confidence interval, p-value, and Bonferroni-adjusted p-value for each of the 53 procedures. All estimates reflect a 1 µg/m^3^ increase in PM_2.5_ and all p-values that were Bonferroni significant (p < 0.05/53) are noted with a *.

**S4 Table. Primary and sensitivity analysis comparison for the three Bonferroni significant procedures**

|  | Primary quasi-Poisson | | quasi-Poisson with adjustment for access to Healthcare and Healthy Foods | | Poisson Mixed-Effects Model | | | Zero-Inflated Poisson Model | | PM < 12 µg/m^3^ | | |  |
| --- | --- | --- | --- | --- | --- | --- | --- | --- | --- | --- | --- | --- | --- |
| Procedure | **Percent Change** | **95% Confidence Interval** | **Percent Change** | **95% Confidence Interval** | **Percent Change** | **95% Confidence Interval** | **Percent Change** | | **95% Confidence Interval** | | **Percent Change** | **95% Confidence Interval** | |
| Glycosylated Hemoglobin Test | 10.8% | 6.56%; 15.1% | 0.03% | -0.42%; 0.49% | 9.45% | 5.27%; 13.8% | 16.0% | | 11.4%; 20.8% | | 5.98% | 1.33%; 10.8% | |
| Prothrombin Time Test | 15.8% | 9.07%; 22.9% | 1.46% | 0.71%;  2.22% | 12.0% | 9.26%; 14.7% | 16.2% | | 14.1%; 18.4% | | 15.1% | 8.07%; 22.7% | |
| Stress Tests | 6.84% | 3.65%; 10.1% | -0.04% | -0.33%; 0.25% | 6.87% | 3.42%; 10.4% | 13.3% | | 9.34%;  17.4% | | -2.45% | -6.22%; 1.47% | |

The percent change and 95% confidence interval (per 1 µg/m^3^ increase in PM_2.5_) for the primary (Primary quasi-Poisson) and sensitivity analyses for each of the three procedures that were Bonferroni significant (p < 0.05/53) in the primary analysis. Additionally shown is the analysis restricted to exposures below the current national ambient air quality standards (PM_2.5_ < 12 µg/m^3^).

**Figures**


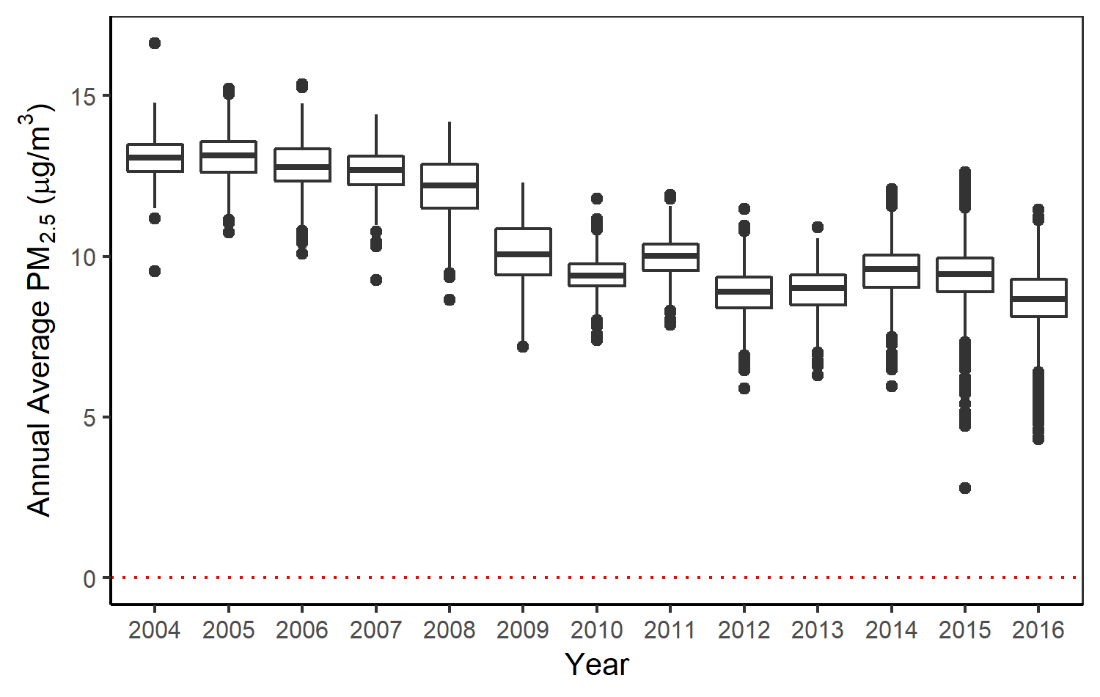


**S1 Fig. PM_2.5_ concentrations each year of the study period.**

Shown here is a box plot of the PM_2.5_ concentrations (μg/m^3^) each year of the study period (2004-2016).


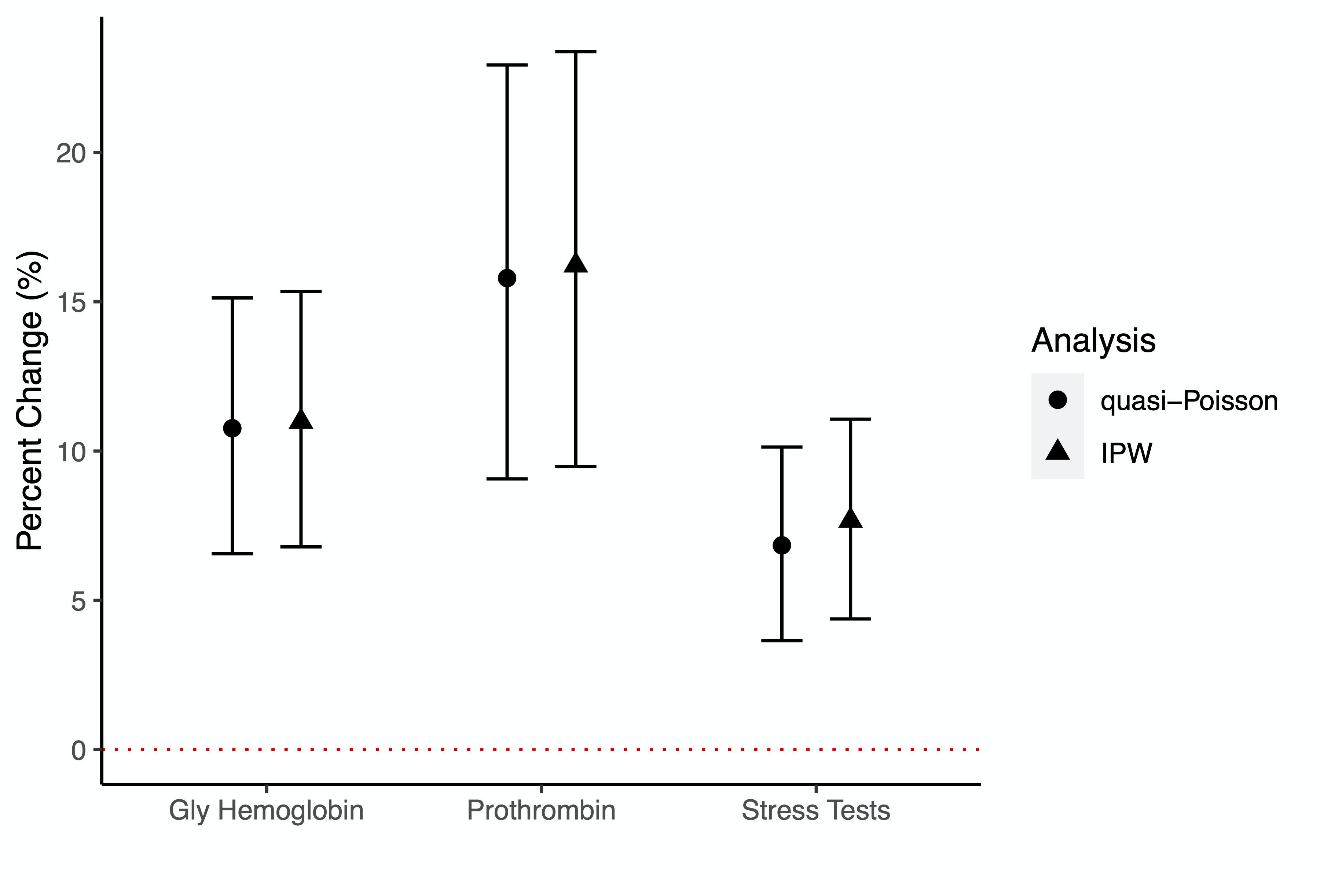


**S2 Fig. Associations between annual average PM_2.5_ and hospital procedures among heart failure patients.**

Shown here are associations between annual average PM_2.5_ and the three Bonferroni significant procedures. Significance was determined according to the primary analysis approach (quasi-Poisson). We also show the percent change and 95% confidence interval for the inverse probability weighted (IPW) model, which adjusted for the competing risk of death. See Methods for complete description of all analysis approaches. Percent change and 95% confidence interval presented per 1 µg/m^3^ increase in PM_2.5_. Gly Hemoglobin = Glycosylated Hemoglobin Test; Prothrombin = Prothrombin Time Test.


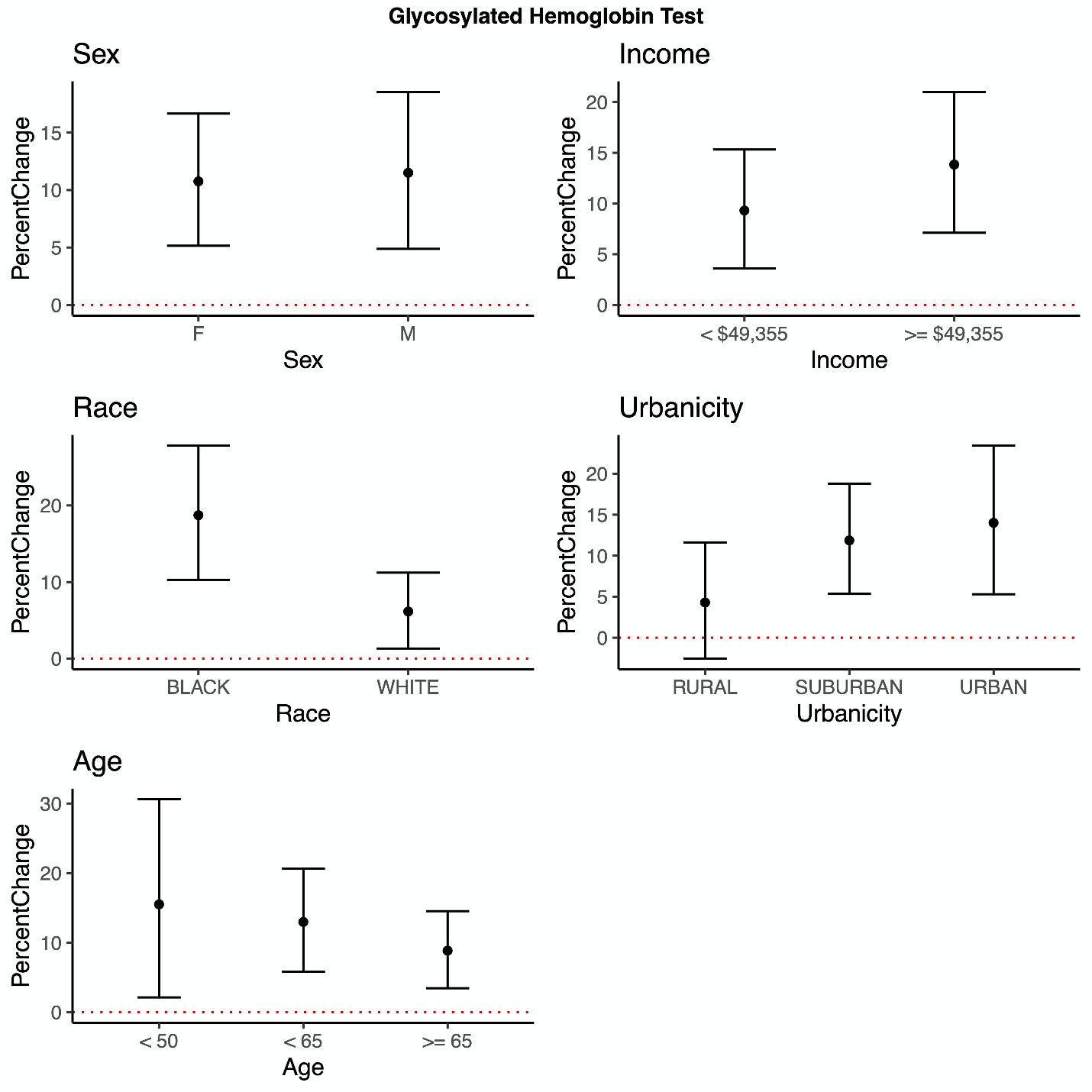


**S3 Fig. Stratified models for Glycosylated Hemoglobin Tests.**

Percent changes and 95% confidence intervals for Glycosylated Hemoglobin Test for each of the following stratified tests: Sex, Median household income, Race, Urbanicity, Age.


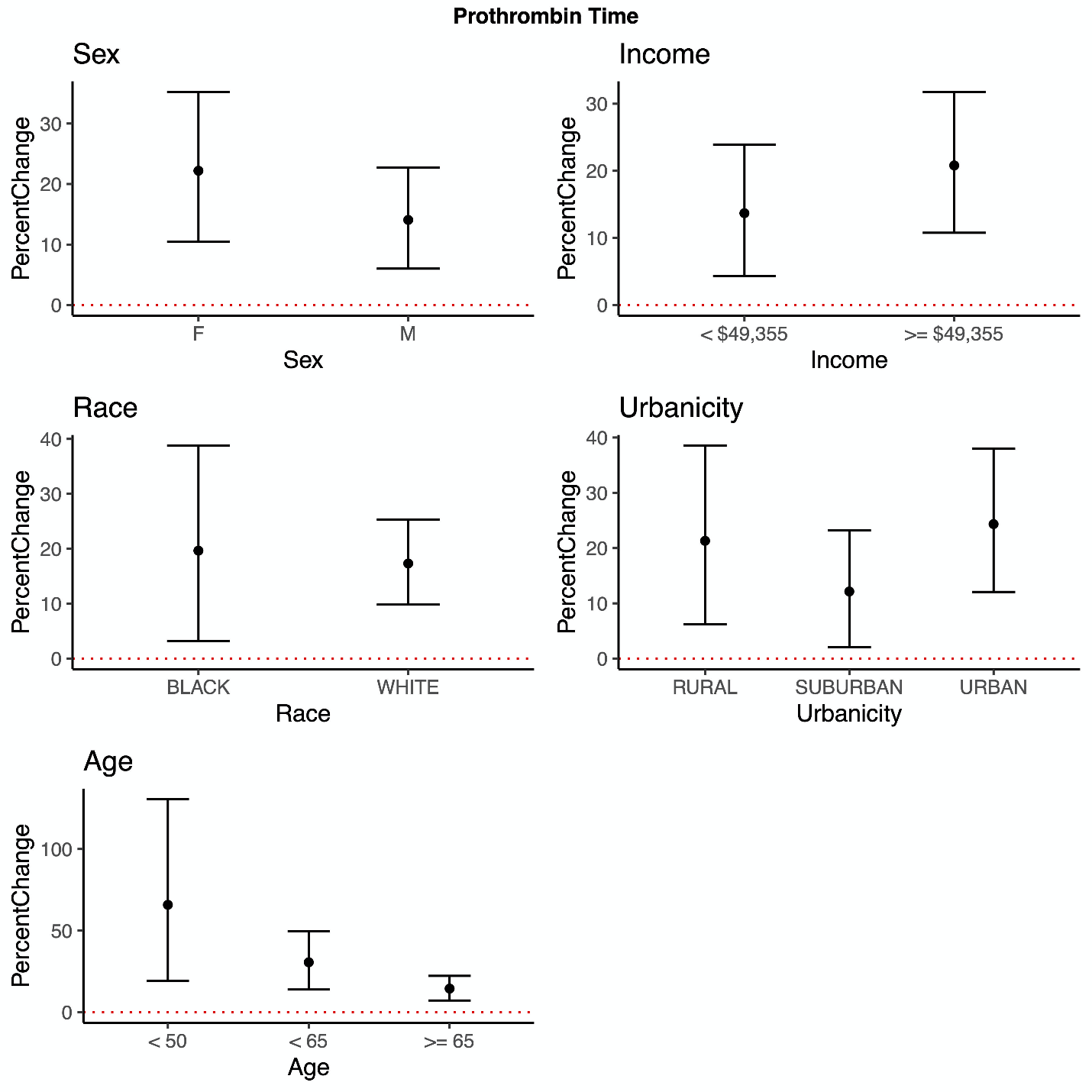


**S4 Fig. Stratified models for Prothrombin Time Tests.**

Percent changes and 95% confidence intervals for Prothrombin Time Test for each of the following stratified tests: Sex, Median household income, Race, Urbanicity, Age.


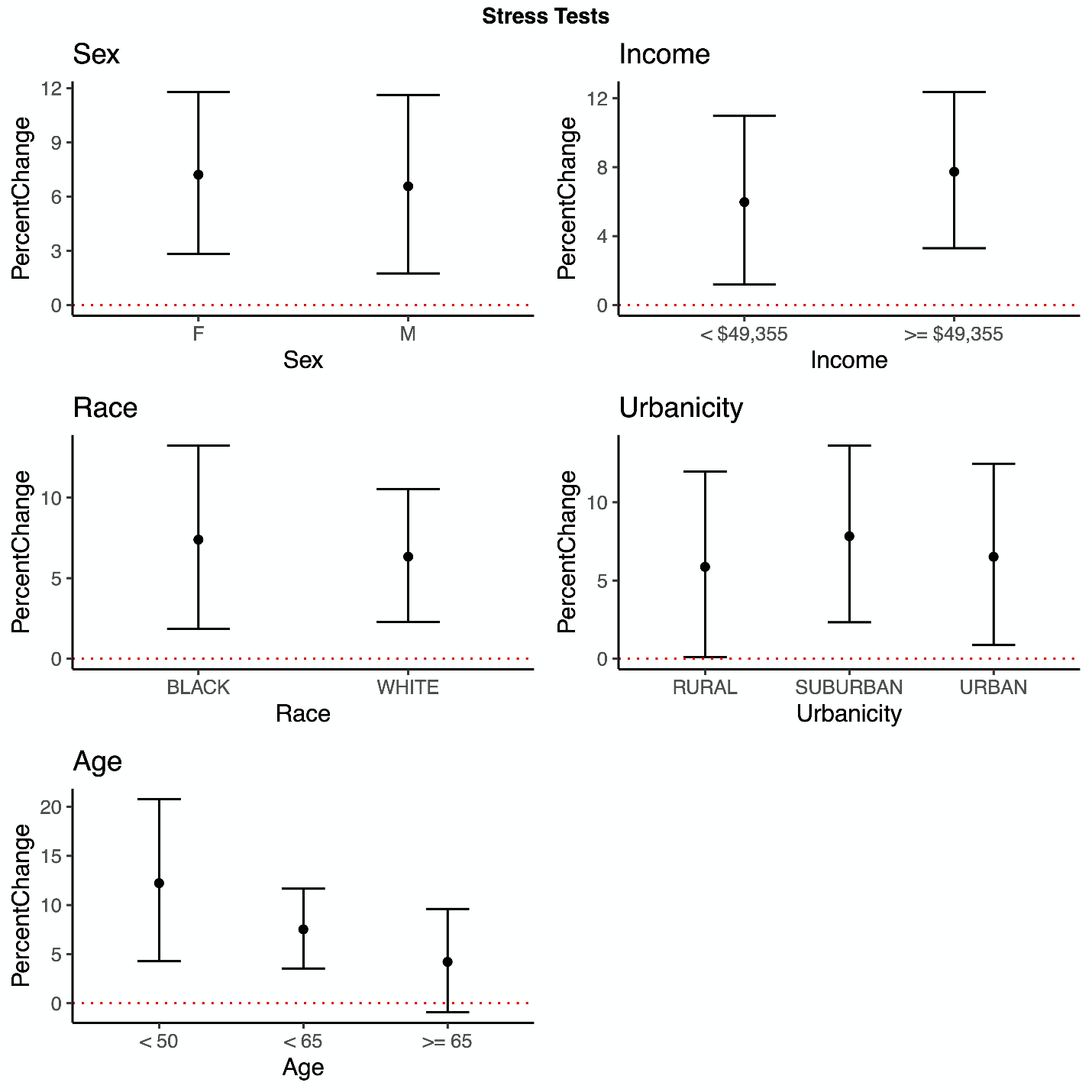


**S5 Fig. Stratified models for Stress Tests.**

Percent changes and 95% confidence intervals for Stress Tests for each of the following stratified tests: Sex, Median household income, Race, Urbanicity, Age.
